# Biofilm Formation and Antibiotic Resistance Genes of *Escherichia coli* From Poultry Farms and Clinical Samples

**DOI:** 10.1002/vms3.70510

**Published:** 2025-07-23

**Authors:** Arina Sasoon, Farhad Nikkhahi, Amir Javadi, Samira Sabzi, Mohadeseh Ostovari Deilamani, Niloofar Kiaheyrati, Amin Karampour, Amir Peymani, Fatemeh Fardsanei

**Affiliations:** ^1^ Medical Microbiology Research Center Qazvin University of Medical Sciences Qazvin Iran; ^2^ Student Research Committee Qazvin University of Medical Sciences Qazvin Iran

**Keywords:** antibiotics resistance, biofilm formation, *Escherichia coli*, poultry

## Abstract

**Background:**

*Escherichia coli* affects human health through intestinal and extraintestinal infections. Avian pathogenic *E. coli* (APEC) contributes to colibacillosis in poultry and can develop public health risks. Antibiotic resistance and biofilm‐producer strains are challenges in infection control options.

**Objective:**

This study aimed to characterize phenotypic and genotypic antibiotic resistance profiles as well as biofilm formation assay in *E. coli* isolates from clinical and poultry samples.

**Methods:**

In the study, 42 *E. coli* isolates were collected and confirmed from clinical and poultry sources. The isolates were evaluated for pathotypes using polymerase chain reaction (PCR). Antibiotic resistance was evaluated using the disk diffusion technique and minimum inhibitory concentration (MIC) tests. PCR was utilized to identify antimicrobial resistance genes associated with fluoroquinolones, sulphonamides, tetracyclines and beta‐lactams. Biofilm formation was evaluated using a 96‐well microtiter plate.

**Results:**

Three clinical isolates, including enteropathogenic *E. coli* (EPEC), enteroaggregative *E. coli* (EAEC) and enterotoxigenic *E. coli* (ETEC), were identified as pathogenic strains. The highest rates of resistance were recorded against tylosin (100%), neomycin (92.85%), tetracycline (85.7%), ampicillin (73.8%), doxycycline (71.4%), ciprofloxacin (64.28%), trimethoprim/sulfamethoxazole (64.28%) and enrofloxacin (57.1%). The most prevalent resistance genes detected as *blaTEM* and *gyrA*/*B* (97.6% and 76.1%, respectively). The overall prevalence of *blaCTX, sul1, sul2, tetA* and *tetB* genes were 21.4%, 45.2%, 11.9%, 33.3% and 7.1%, respectively. The *qnrB, qnrB4* and *qnrS* genes were absent in the clinical samples, whereas present in poultry isolates. All isolates were biofilm producers, and 96.4% of poultry isolates had strong biofilm formation capacity.

**Conclusion:**

The alarming levels of resistance genes and biofilm formation of isolates in the present study emphasize the need for antibiotic management practices and further research on resistance transmission dynamics in the food industry.

## Graphical abstract

Three clinical isolates, including EPEC, EAEC and ETEC, were identified as pathogenic strains. All isolates were biofilm producers, and 96.4% of poultry isolates had strong biofilm formation capacity. The highest rates of resistance were recorded against tylosin (100%), neomycin (92.85%), tetracycline (85.7%), ampicillin (73.8%), doxycycline (71.4%), ciprofloxacin (64.28%), trimethoprim/sulfamethoxazole (64.28%) and enrofloxacin (57.1%).

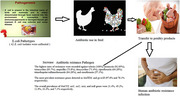

## 1 Introduction

*Escherichia coli (E. coli)*, a multifaceted facultative anaerobic bacteria, is a commensal resident of the intestines in humans and animals (Foster‐Nyarko and Pallen 2022). This microorganism is commonly classified on the basis of its pathogenicity into nonpathogenic, intestinal pathogenic and extraintestinal pathogenic strains. In healthcare settings, *E. coli* is a well‐known pathogen that causes various infections, from urinary tract infections (UTIs) to more severe conditions like bloodstream infections and gastrointestinal diseases (Vihta et al. 2018). Intestinal pathogenic *E. coli* is classified into various pathogenic groups on the basis of their virulence genes. These pathotypes include enterohemorrhagic *E. coli* (EHEC), enteropathogenic *E. coli* (EPEC), enteroinvasive *E. coli* (EIEC), enterotoxigenic *E. coli* (ETEC) and enteroaggregative *E. coli* (EAEC) (Pokharel et al. 2023). ETEC is commonly found in low‐ and middle‐income countries and is a significant cause of travellers’ diarrhoea, especially in children in these regions. EAEC is often linked to a higher risk of persistent diarrhoea. At the same time, EPEC is related to long‐lasting diarrhoea in developing countries and may also play a role in causing diarrhoea in more developed nations (Gomes et al. 2016).

Avian pathogenic *E. coli* (APEC) is accounting for causing colibacillosis in chicken and poultry, leading to extraintestinal infections (Cunha et al. 2017). Avian colibacillosis manifests in various forms, such as acute septicaemia, chronic respiratory diseases, cellulitis, salpingitis, pericarditis and other extraintestinal diseases (Panth 2019). Pathogenic *E. coli* isolates in animals and birds served as reservoirs, facilitating the spread of these strains among different livestock (Yuan et al. 2021). This can lead to contamination of the environment and increase the risk of infection in humans through direct exposure and consumption of contaminated food or water (Schmithausen et al. 2018).

The development of antibiotic resistance in gram‐negative bacteria, particularly in *E. coli*, poses a serious threat to global public health. *E. coli* is commonly used as an indicator for monitoring antibiotic resistance in the environment, as it has shown a strong capability to acquire multiple antibiotic resistances (Anjum et al. 2021). There are concerns about the transmission of antibiotic‐resistant bacteria from animals to humans due to the widespread use of antibiotics in animals for prevention and growth promotion (Aworh et al. 2021; Osman et al. 2018). Increasing antibiotic resistance among foodborne bacteria like *E. coli* has resulted in higher rates of human fatalities, longer hospital stays and increased healthcare costs (Mathers et al. 2015).

Biofilms are coatings produced by microorganisms that adhere to surfaces, creating protective layers as a survival strategy (Li et al. 2023). Biofilm has been recognized as a significant contributor to the development of antimicrobial resistance and serves as a protective barrier that prevents the effective targeting of microorganisms in living tissues by antimicrobial agents, antibodies, and immune cells (Kumar et al. 2017). APECs in poultry water systems are survived by biofilm, which is present on all equipment used in food production, including waterers and pipes used for drinking (Gunathilaka et al. 2024). This study aims to determine and characterize the antibiotic resistance patterns, and formation of bacterial biofilms in *E. coli* strains obtained from both clinical samples and poultry infected with colibacillosis.

## 2 Materials and Methods

### 2.1 Sampling and Bacterial Isolation

A total of 42 *E. coli* strains were identified in samples collected from humans and poultry from April to September 2022 in Qazvin, Iran. Out of these, 28 strains were isolated from the liver and intestine samples of poultry infected with colibacillosis, and 14 strains were obtained from clinical samples. Enrichment was performed using peptone water and Rappaport Vassiliadis for poultry samples, and the stool samples were transferred into gram‐negative broth (GN Broth) incubated at 37°C for 8 h. Then, the cultures were again sub‐cultured on EMB (eosin methylene blue) and XLD (xylose lysine deoxycholate) agar. Suspected colonies were subjected to standard biochemical tests, including oxidase, catalase, TSI agar, methyl red‐Voges–Proskauer (MRVP), citrate consumption (Simmons citrate agar) and urease production. All isolates that were glucose positive, lactose negative, MR positive, VP negative, indole positive, motility positive, hydrogen sulphide negative, and citrate negative were considered *E. coli* isolates by phenotypic tests. Stocks of all isolates were stored frozen at −80°C in microtube containing Tryptic Soy Broth (TSB) complemented with 10% (v/v) glycerol until required. Bacteria were recovered from freezing by transferring 100 µL into 5 mL of TSB, incubating overnight at 37°C, and sub‐culturing prior to use in assays.

### 2.2 Molecular Confirmation of *E. coli* Strains

#### 2.2.1 DNA Extraction

*E. coli* isolates were cultured overnight on Trypticase soy agar (Sigma Aldrich, USA) plates, followed by the extraction of genomic DNA using the boiling method (da Silva et al. 2012). Briefly, bacteria were suspended in double‐distilled water and lysed by heating at 100°C for 10 min. After centrifugation, the supernatant containing DNA was collected and stored at −20°C until polymerase chain reaction (PCR) analysis.

#### 2.2.2 PCR Assay

The PCR assay was conducted to detect different pathotypes of diarrheagenic *E. coli* strains by targeting virulence genes related to these strains. The specific primer sequences (Havt et al. 2017) are listed in Table 1. The pathotypes tested included EHEC (*stx1, stx2* and *hlyA* genes), ETEC (*lt* and *st* genes), EAEC (*aggR, sigA* and *astA* genes) and EPEC (*eae* gene). The PCR products were separated by electrophoresis on a 1% agarose gel.

**TABLE 1:** Primer sequences used for the identification of different *Escherichia coli* pathotypes by polymerase chain reaction (PCR).

Pathotype	Target gene	Primer sequence (5′–3′)	PCR product (bp)
EHEC	*Stx1*	F: ATGTCATTCGCTCTGCAATAGGTAC	1020
		R: GAAGAAGAGACTGAAGATTCCATCTG	
EHEC	*Stx2*	F: GGCACTGTCTGAAACTGCTCCTGT	625
		R: ATTAAACTGCACTTCAGCAAATCC	
EHEC	*hlyA*	F: AGCTGCAAGTGCGGGTCTG	569
		R:TACGGGTTATGCCTGCAAGTTCAC	
EPEC	*eae*	F: ATGCTTAGTGCTGGTTTAGG	248
		R: GCCTTCATCATTTCGCTTTC	
ETEC	*st*	F: AGGAACGTACATCATTGCCC	170
		R: CAAAGCATGCTCCAGCACTA	
ETEC	*lt*	F: GGCGAC AGA TTATACCGTGC	450
		R: CGG TCT CTA TAT TCC CTG TT	
EAEC	*aggR*	F: CTAATTGTACAATCGATGTA	430
		R: ATGAAGTAATTCTTGAAT	
EAEC	*sigA*	F: CCGACTTCTCACTTTCTCCCG	430
		R: CCATCCAGCTGCATAGTGTTTG	
EAEC	*astA*	F: ATGCCATCAACACAGTATAT	110
		R: GCGAGTGACGGCTTTGTAGT	

Abbreviations: EAEC, enteroaggregative *E. coli*; EHEC, enterohemorrhagic *E. coli*; ETEC, enterotoxigenic *E. coli*.

### 2.3 Antibiotic Susceptibility Testing

The disk diffusion method, as per the Clinical & Laboratory Standards Institute guidelines (CLSI 2023), was used to test the susceptibility of isolates to various antibiotics. Various antibacterial discs (Thermo Fisher Scientific, USA), including cefepime (20 µg), cefotaxime (30 µg), ceftazidime (30 µg), cefoxitin (5 µg), neomycin (30 µg), ciprofloxacin (5 µg), lincospectin (30 µg), tylosin (40 µg), danofloxacin (40 µg), Tetracycline (30 µg), Enrofloxacin (10 µg), Doxycycline (30 µg), Trimethoprim/Sulfamethoxazole (25 µg), imipenem (10 µg), ampicillin (30 µg), florfenicol (30 µg) and levofloxacin (5 µg), were selected and tested. In addition, the minimum inhibitory concentration (MIC) of colistin was determined. Extended Spectrum Beta‐Lactamase (ESBL)‐producing isolates were identified using phenotypic methods as described previously (Rupp and Fey 2003). *E. coli* ATCC 25922 served as the standard strain for comparison.

### 2.4 Antimicrobial Resistance Gene Identification

PCRs with specific primers were applied to evaluate the presence of 14 antibiotic‐resistant genes among different classes of antibiotics. Isolates were screened for fluoroquinolone resistance: *qnrA, qnrB, qnrB4, qnrC, qnrD, qnrS, gyrA* and *gyrB*; sulfonamide resistance: *sul1, sul2* and *sul3*; tetracyclines: *tetA*, *tetB* and *tetG*; and beta‐lactams resistance: *blaCTX‐M* and *blaTEM*. Table 2 provides a list of the primer sequences used in the study along with the corresponding annealing temperatures. The PCR products were resolved on a 1% agarose gel and visualized using a UV‐trans illuminator.

**TABLE 2:** Primer sequences applied for resistance gene identification.

Antibiotic group	Target gene	Primer sequence (5′–3′)	Annealing (°C)	Reference
Fluoroquinolone	*qnrA*	F: ATTTCTCACGCCAGGATTTG	53	Dallal et al. (2023)
		R: GATCGGCAAAGGTTAGGTCA		
	*qnrB*	F:GATCGTGAAAGCCAGAAAGG	53	Dallal et al. (2023)
		R: ACGATGCCTGGTAGTTGTCC		
	*qnrB4*	F: AGTTGTGATCTCTCCATGGC	53	Rezazadeh et al. (2016)
		R: CGGATATCTAAATCGCCCAG		
	*qnrC*	F: GGGTTGTACATTTATTGAATC	57	Abdel‐Rhman et al. (2021)
		R: TCCACTTTACGAGGTTCT		
	*qnrD*	F: CGGGGAATAGAGTTAAAAAT	47	Veldman et al. (2011)
		R: TATCGGTGAACAATAACACC		
	*qnrS*	F: GCAAGTTCATTGAACAGGGT	53	Abdel‐Rhman et al. (2021)
		R: TCTAAACCGTCGAGTTCGGCG		
	*gyrA*	F: TGCCAGATGTCCGAGAT	60	Oram and Fisher (1991)
		R: GTATAACGCATTGCC		
	*gyrB*	F: GGCACTGAATTTATCGGC	60	Ahmed et al. (2005)
		R: TCCGAATTGGTCAGATCG		
Sulfonamide	*Sul1*	F:ATGGTGACGGTGTTCGGCATTCTG	64	Dallal et al. (2023)
		R: GCTAGGCATGATCTAACCCTCGG		
	*Sul2*	F: AGGGGGCAGATGTGATCGAC	59	Dallal et al. (2023)
		R: GCAGATGATTTCGCCAATTG		
	*Sul3*	F: TCAAAGCAAAATGATATGAGC	59	Dallal et al. (2023)
		R: TTTCAAGGCATCTGATAAAGAC		
Tetracycline	*tetA*	F: GTAATTCTGAGCACTGTCGC	58	Dallal et al. (2023)
		R: CTGCCTGGACAACATTGCTT		
	*tetB*	F: TTGGTTAGGGGCAAGTTTTG	60	Dallal et al. (2023)
		R: GTAATGGGCCAATAACACCG	65	
	*tetG*	F: AGCAGCCTCAACCATTGCCGAT		Dallal et al. (2023)
		R: GGTGTTCCACTGAAAACGGTCCT		
β‐Lactam	*bla*_CTX‐M_	F: AGGAAGTGTGCCGCTGTATG	53	Dallal et al. (2023)
		R: CTGTCGCCCAATGCTTTACC		
	*Bla*_TEM_	F: TCGCCGCATACACTATTCTC	57	Dallal et al. (2023)
		R: AACTTTATCCGCCTCCATCC		

### 2.5 Biofilm Formation Assay

The formation of biofilms was studied using a 96‐well microtiter plate technique. Bacterial cultures were grown in a TSB medium containing 5% sucrose and then adjusted to a standard concentration (0.5 MFU [McFarland]) before being added to each microplate well. The plate was then incubated at 37°C for 18–24 h. *Pseudomonas aeruginosa* and TSB medium were the positive and negative controls, respectively (Wu et al. 2011). Following three quick washes with phosphate‐buffered saline (PBS), and cell fixation with methanol 95% solution, the plate was stained with 200 µL of 1% crystal violet (CV) for 15 min. The dye was taken out, and the wells were rinsed three times with PBS. Subsequently, the biofilms formed were dissolved with 200 µL of 33% acetic acid for 30 min. A microtiter plate reader (BioTek, Epoch, USA) was used to measure the optical absorbance at 570 nm (OD570, ODC570). Biofilms were categorized into four groups on the basis of the ODc value: OD ≤ ODc (non‐biofilm formation), ODc < OD ≤ 2 ODc (weak‐biofilm formation), 2 ODc < OD ≤ 4 ODc (medium‐biofilm formation) and OD > 4 ODc (strong‐biofilm formation).

### 2.6 Statistical Analysis

SPSS software version 16 was used for conducting statistical analysis. The Kruskal–Wallis test was applied for comparisons of biofilm formation capacity. A result was considered to have statistical significance if the *p* value was less than 0.05 (*p* < 0.05).

## 3 Results

### 3.1 Molecular Confirmation of *E. coli* Isolates

Out of the 42 *E. coli* strains, three clinical isolates, including one EPEC, one ETEC and one EAEC, were identified on the basis of the presence of the target genes. The remaining isolates did not exhibit virulence genes characteristic of the tested pathotypes.

### 3.2 Characteristics of Isolates

The highest rates of resistance were recorded against tylosin (*N* = 42, 100%), neomycin (*N* = 39, 92.85%), tetracycline (*N* = 36, 85.7%), ampicillin (*N* = 31, 73.8%), doxycycline (*N* = 30, 71.4%), ciprofloxacin (*N* = 27, 64.28%) and trimethoprim/sulfamethoxazole (27, 64.28%). The resistance rates to other antibiotics were as follows: enrofloxacin (*N* = 24, 57.1%), danofloxacin (*N* = 20, 47.6%), levofloxacin (*N* = 19, 45.2%), florfenicol (*N* = 18, 42.8%), cefotaxime (*N* = 15, 35.7%), imipenem (*N* = 8, 19%), lincospectin (*N* = 7, 16.6%), ceftazidime (*N* = 5, 11.9%), cefoxitin (*N* = 5, 11.9%) and cefepime (*N* = 2, 4.7%). Additionally, all 42 *E. coli* isolates in the study exhibited MIC ≤ 2 to colistin. The CLSI no longer categorizes *E. coli* as colistin‐susceptible, only recognizing intermediate (MIC ≤ 2) and resistant (MIC ≥ 4) categories. However, the EUCAST 2023 still considers isolates with a colistin MIC ≤ 2 to be colistin‐susceptible (Zafer et al. 2023). Moreover, 11 strains (26.1%) were identified as ESBL positive on the basis of the phenotypic method.

Regarding antibiotic resistance genes, *qnrA, qnrC, qnrD, sul3 and tetG* were not found in any isolates. Among fluoroquinolone‐resistance isolates, the highest resistance was attributed to *gyrA* and *gyrB* genes among both clinical and poultry isolates (overall 76.1%, 85.7% of poultry and 57.1% of clinical isolates). The *qnrB*, *qnrB4* and *qnrS* genes were found in 17.8%, 14.2% and 10.7% of poultry isolates, respectively, but not in clinical isolates.

Among sulphonamides resistance isolates, 42.8% and 46.4% of clinical and poultry isolates were positive with *sul1* gene. The *sul2* gene was found in 10.7% and 14.2% of poultry and clinical isolates, respectively.

In terms of beta‐lactam resistance, the most prevalent resistance gene identified was *blaTEM*, present in 96.4% of poultry and 100% of clinical isolates. The *blaCTX* gene was also detected in 17.8% of poultry isolates and 28.5% of clinical isolates.

Tetracycline resistance was linked to the *tetA* gene in 32.1% of poultry isolates and 14.2% of clinical isolates. The *tetB* gene was found in 7.1% of both poultry and clinical isolates, whereas the *tetG* gene was not identified in any isolates. The detailed information is presented in Table 3.

**TABLE 3:** Phenotypic and genotypic characterization of *Escherichia coli* isolates from clinical and poultry samples.

Isolates	Source	Phenotypic resistance	Genotypic resistance	Biofilm formation
SE1	Poultry	N, TY, T, CP, D, AMP	*gyrA*, *gyrB*, *bla*_TEM_	Strong
SE2	Poultry	N, TY, T, D, AMP	*bla*_TEM_	Strong
SE3	Poultry	N, FF, TY, DFX, T, CP, NFX, D, LEV, AMP	*gyrA*, *gyrB*, *bla*_TEM_, *qnrB*	Strong
SE4	Poultry	N, TY, T, CP, ENR, D, AMP	*gyrA*, *gyrB*, *bla*_TEM_ *, tetA*	Strong
SE5	Poultry	N, TY, AMP	*bla*_TEM_	Strong
SE6	Poultry	N, TY, AMP	*qnrB, qnrB_4_, bla*_TEM_	Strong
SE7	Poultry	N, FF, TY, DFX, CP, ENR, SXT, LEV	*gyrA*, *gyrB*, *bla*_TEM_, *qnrB, qnrB_4_*	Strong
SE8	Poultry	N, TY, DFX, T, CP, ENR, D, SXT, LEV	*gyrA*, *gyrB*, *bla*_TEM_ *, tetA, sul1*	Strong
SE9	Poultry	CTX, CAZ, FOX, N, FF, TY, DFX, T, CP, ENR, D, SXT, LED, AMP	*gyrA*, *gyrB*, *bla*_TEM_ *, tetA, sul1, qnrB, qnrB_4_*	Strong
SE10	Poultry	N, FF, TY, DFX, CP, ENR, SXT, LEV, AMP	*gyrA*, *gyrB*, *bla*_TEM_ *, tetA, sul1, qnrB, qnrB_4_*	Strong
SE11	Poultry	CTX, N, LS, FF, TY, T, CP, ENR, SXT, AMP	*gyrA*, *gyrB, bla*_TEM_ *, bla*_CTX‐M_, *tetA, sul1*	Strong
SE12	Poultry	CTX, N, LS, FF, TY, T, CP, ENR, SXT, AMP	*gyrA*, *gyrB*, *bla*_TEM_, *bla*_CTX‐M_, *sul2*	Strong
SE13	Poultry	CTX, N, LS, FF, TY, DFX, T, CP, ENR, D, SXT, LEV, AMP	*gyrA*, *gyrB*, *bla*_TEM_, *bla*_CTX‐M_, *tetA, sul1*	Strong
SE14	Poultry	N, TY, T, CP, D, SXT	*gyrA*, *gyrB*, *bla*_TEM_ *, tetA, sul2, qnrS*	Strong
SE15	Poultry	CTX, N, TY, DFX, T, CP, ENR, D, SXT, LEV, AMP	*gyrA*, *gyrB*, *bla*_TEM_ *, sul1, qnrS*	Strong
SE16	Poultry	CTX, N, FF, TY, DFX, T, CP, ENR, D, SXT, LEV, AMP, IMP	*gyrA*, *gyrB*, *bla*_TEM_ *, bla*_CTX‐M_, *sul1*	Strong
SE17	Poultry	CTX, TY, DFX, T, CP, ENR, D, SXT, LEV	*gyrA*, *gyrB*, *bla*_CTX‐M_, *tetA, sul1*	Strong
SE18	Poultry	FOX, N, FF, TY, DFX, T, CP, ENR, D, SXT, LEV, AMP	*gyrA*, *gyrB*, *bla*_TEM_ *, sul1*	Strong
SE19	Poultry	N, FF, TY, DFX, T, CP, ENR, D, LEV, AMP	*gyrA*, *gyrB*, *bla*_TEM_	Strong
SE20	Poultry	N, FF, TY, DFX, T, CP, ENR, D, LEV, AMP	*gyrA*, *gyrB*, *bla*_TEM_	Strong
SE21	Poultry	N, FF, TY, DFX, T, CP, ENR, D, LEV, AMP	*gyrA*, *gyrB*, *bla*_TEM_ *, tetB*	Strong
SE22	Poultry	N, FF, TY, DFX, T, CP, NFX, D, SXT, AMP	*gyrA*, *gyrB*, *bla*_TEM_ *, tetB, sul1*	Strong
SE23	Poultry	FF, TY, DFX, T, CP, ENR, D, SXT, LEV, AMP	*gyrA*, *gyrB*, *bla*_TEM_ *, tetA, suL2*	Strong
SE24	Poultry	N, TY, T, CP, D, SXT, LEV, AMP	*gyrA*, *gyrB*, *bla*_TEM_ *, sul1*	Strong
SE25	Poultry	FOX, N, FF, TY, DFX, T, CP, ENR, D	*gyrA*, *gyrB*, *bla*_TEM_ *, tetA, sul1*	Strong
SE26	Poultry	N, FF, TY, DFX, T, CP, ENR, D, SXT, LEV	*bla*_TEM_ *, sul1*	Strong
SE27	Poultry	N, TY, DFX, T, CP, ENR, D, SXT, LEV, AMP	*gyrA*, *gyrB*, *bla*_TEM_	Moderate
SE28	Poultry	N, TY, T, ENR, D, SXT	*gyrA*, *gyrB*, *bla*_TEM_, *qnrS*	Strong
SE29	Clinical	CTX, N, FF, TY, T, D, SXT, AMP	*bla*_TEM_ *, sul1*	Moderate
SE30	Clinical	CTX, N, TY, T, D, SXT, AMP	*bla*_TEM_ *, sul2*	Strong
SE31	Clinical	N, TY, T, D	*bla*_TEM_	Strong
SE32	Clinical	TY, T	*bla*_TEM_	Weak
SE33	Clinical	N, TY, CP, SXT, AMP, IMP	*gyrA*, *gyrB*, *bla*_TEM_ *, sul1*	Strong
SE34	Clinical	CTX, CAZ, N, LS, TY, T, D, SXT, AMP	*gyrA*, *gyrB*, *bla*_TEM_ *, bla*_CTX‐M_, *tetB, sul2*	Moderate
SE35	Clinical	CTX, CAZ, N, LS, FF, TY, T, SXT, AMP, IMP	*bla*_TEM_ *, bla*_CTX‐M_	Strong
SE36	Clinical	FOX, N, TY	*gyrA*, *gyrB*, *bla*_TEM_	Weak
SE37	Clinical	FEP, CTX, CAZ, FOX, N, LS, TY, DFX, T, CP, ENR, D, SXT, LEV, AMP, IMP	*gyrA*, *gyrB*, *bla*_TEM_ *, bla*_CTX‐M_ *, tetA, sul1*	Moderate
SE38	Clinical	N, TY, T, D, SXT, AMP, IMP	*gyrA*, *gyrB*, *bla*_TEM_ *, tetA, sul1*	Strong
SE39	Clinical	FEP, N, TY, T, AMP	*gyrA*, *gyrB*, *bla*_TEM_	Strong
SE40	Clinical	CTX, CAZ, N, LS, TY, T, CP, D, SXT, AMP, IMP	*gyrA*, *gyrB*, *bla*_TEM_ *, bla*_CTX‐M_, *sul1*	Moderate
SE41	Clinical	CTX, N, TY, T, IMP	*bla*_TEM_	Moderate
SE42	Clinical	CTX, N, TY, DFX, T, CP, ENR, D, SXT, LEV, AMP, IMP	*gyrA*, *gyrB*, *bla*_TEM_ *, sul1*	Weak

Abbreviations: Amp, ampicillin; CAZ, ceftazidime; CP, ciprofloxacin; CTX, cefotaxime; D, doxycycline; DFX, danofloxacin; ENR, enrofloxacin; FEP, cefepime; FF, florfenicol; IMP, imipenem; LEV, levofloxacin; LS, lincospectin; N, neomycin; SXT, trimethoprim/sulfamethoxazole; T, tetracycline; TY, tylosin.

### 3.3 Biofilm Formation Assay

**FIGURE 1:**
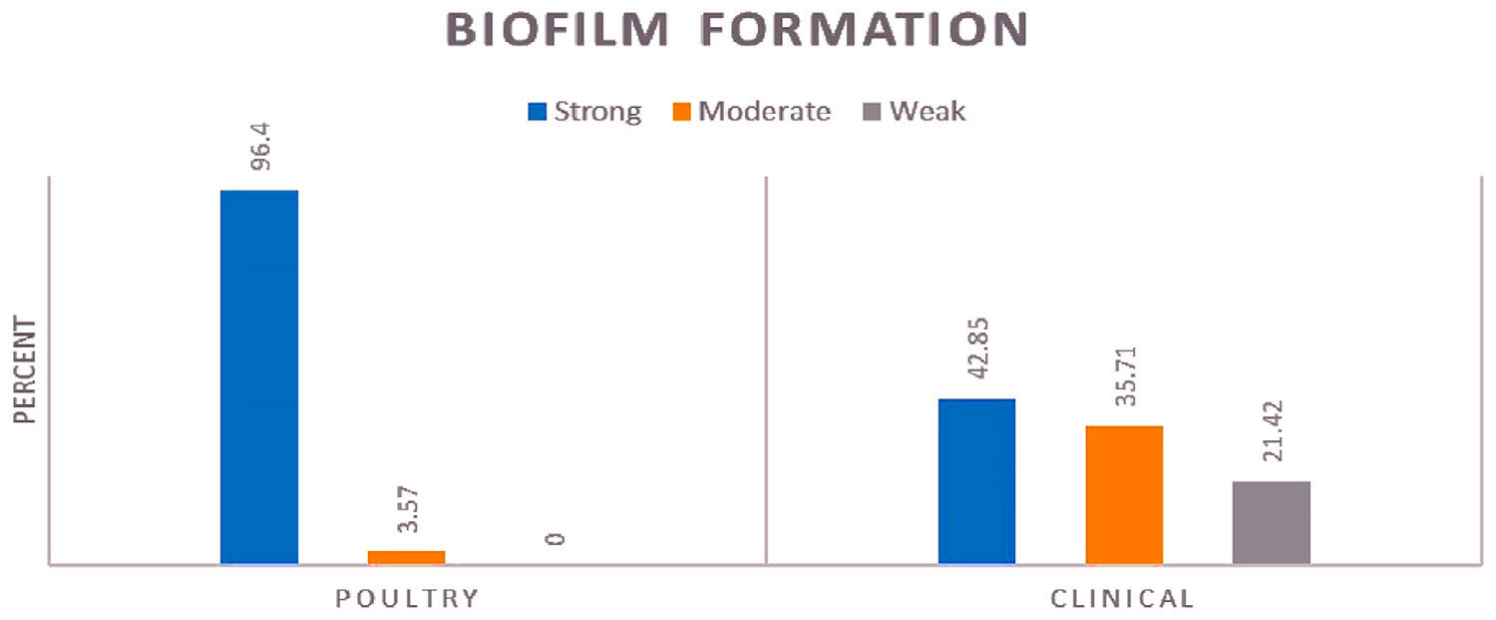
Comparison of biofilm producer's strains in clinical and poultry of *Escherichia coli*.

The findings showed that every isolated tested could form biofilm. Among clinical isolates, 21.42% were categorized as weak biofilm producers, 35.71% as moderate biofilm producers, and the majority, accounting for 42.85%, were identified as strong biofilm producers. Compared to isolates taken from clinical samples, a significant majority (96.4%) of isolates from poultry samples demonstrated the ability to form robust biofilm, and only 3.57% of isolates formed medium biofilm. None of the poultry isolates formed a weak biofilm. The distribution of biofilm formation according to sample sources is visually represented in Figure 1 and Table 3. There was no association between biofilm formation or antibiotic resistance patterns and resistance genes.

## 4 Discussion

Pathogenic strains of *E. coli* can be transmitted to a host through various pathways, particularly the faecal‐oral route. These virulent strains can readily contaminate the human food chain, leading to the development of severe gastrointestinal illnesses (Alia et al. 2022; Sharifi Yazd et al. 2011). This study identified three clinical *E. coli* isolates as EPEC, ETEC and EAEC, highlighting the presence of these virulent pathotypes in Iran. In a study conducted by Eybpoosh et al. (2021), it was found that ETEC and EPEC were the second and third most commonly found pathotypes in Iran, whereas EAEC did not show significant prevalence.

The AST conducted in this study revealed that many identified isolates had significant levels of resistance to multiple antibiotics. The highest resistance rate was identified for tylosin (100%) and neomycin (92.8%). The widespread use of tylosin and neomycin in animal feed for promoting growth and preventing illness could be a contributing factor to the high levels of resistance to these antibiotics (Driver et al. 2009; Schmidt et al. 2020).

Quinolones are frequently applied to treat various infections caused by *E. coli* and other bacteria in the Enterobacteriaceae family. However, the emergence of resistance to these antibiotics has made treatment more challenging and could result in ineffective therapy (Bush et al. 2020). In the present study, 64.2%, 57.1%, 47.6% and 45.2% of isolates were fully resistant to ciprofloxacin, enrofloxacin, danofloxacin and levofloxacin, respectively. These results were higher than those found in past studies conducted in China (Cheng et al. 2020) and Iran (Hamed et al. 2021). Furthermore, in the molecular investigations, among the PMQR (plasmid‐mediated quinolones resistance) determinants, *qnrB*, *qnrB4* and *qnrS* genes were identified only in poultry isolates. This finding suggested the spread of quinolone‐resistant *E. coli* from food‐producing animals to humans cannot be ignored, as *qnr* can be transferred to pathogenic bacteria through commensal *E. coli*. Our finding is consistent with Tamang et al. (2012) from Korea and Yue et al. (2008) from China.

In our study, resistance to ampicillin was prevalent (overall 73.8%), whereas resistance rates to other beta‐lactams, including ceftazidime, cefoxitin and cefepime, were uncommon among isolates. These findings align with previous research (Adenipekun et al. 2016; Dallal et al. 2023). Consideringly, 19% of all *E. coli* isolates determinate resistance to imipenem. Resistance to imipenem can pose a serious threat, as this antibiotic may be the last resort for the treatment of severe infections caused by pathogenic bacteria (Sharma et al.). The molecular results showed that *blaTEM* was the most common ESBL gene found in isolates (100% for clinical isolates and 96.4% for poultry isolates), which aligns with what other studies have found (Dallal et al. 2023; Reshadi et al. 2021). Some studies found chickens exhibited a higher frequency of the *blaTEM* gene, possibly due to the increased application of beta‐lactam antibiotics in poultry farms (Ogunrinu et al. 2020; Saliu et al. 2017). Furthermore, the *blaCTX‐M* gene was found in 17.9% of the poultry isolates and 28.6% of clinical samples. The prevalence of this gene has shown an increase compared to a recent study conducted in Iran (Pourhossein et al. 2020) but decreased compared to the study in China (Li et al. 2016).

Tetracyclines and sulphonamides are essential types of antibiotics commonly used on their own or in combination with other antibiotics to treat infections caused by Enterobacteriaceae (Gao et al. 2012). The high levels of tetracycline (overall 85.7%), doxycycline (overall 71.4%) and Trimethoprim/sulfamethoxazole (overall 64.28%) among isolates are concerning. Interestingly, this study found that tetracycline and doxycycline resistance in poultry sources was more common than in clinical sources. This suggests the significance of closely monitoring and regulating the use of antibiotics in poultry farms to control the transmission of antibiotic‐resistant bacteria to humans via food supplies. Consistent with previous research, our findings indicate that *tetA* was the most common determinant (Jahantigh et al. 2020; Soltan Dallal et al. 2023). However, Skocková et al. (2012) found *tetB* as the most prevalent tetracycline resistance gene.

Biofilm formation characteristics among *E. coli* isolates are another crucial aspect highlighted in this study. The high proportion of isolates capable of forming strong biofilms indicates the adaptability and persistence of these bacteria in diverse environments. On the basis of the results, biofilm formation was detected in all tested isolates using the microtiter plate method and was more prevalent than previously reported (Pavlickova et al. 2017; Rodrigues et al. 2019). Our results indicate a significant presence of biofilm‐forming strains in poultry, demonstrating strong genotypic and phenotypic resistance profiles to various antibiotics. The dominance of strong biofilm producers in poultry isolates of this study (nearly 97%) warrants further investigation into the role of agricultural practices in biofilm development and transmission dynamics (Hussain et al. 2017; Siddique et al. 2021). Future research endeavours should concentrate on understanding the mechanisms underlying these resistance patterns and developing novel strategies to combat biofilm‐associated antibiotic resistance.

## 5 Conclusion

This research offers a deeper understanding of the frequency and features of *E. coli* strains found in human and poultry samples in Qazvin, Iran. The co‐occurrence of high antimicrobial resistance and strong biofilm‐forming capabilities in strains highlights the urgent need for coordinated efforts to combat antibiotic resistance and ensure the effectiveness of antimicrobial therapies.

## Author Contributions

**Arina Sasoon**: methodology, investigation, visualization. **Farhad Nikkhahi**: supervision, visualization. **Amir Javadi**: formal analysis, software. **Samira Sabzi**: writing – original draft. **Mohadeseh Ostovari Deilamani**: methodology. **Niloofar Kiaheyrati**: writing – review and editing. **Amin Karampour**: project administration. **Amir Peymani**: writing – review and editing. **Fatemeh Fardsanei**: supervision, writing – review and editing, data curation, writing – original draft, validation, funding acquisition, resources, conceptualization.

## Ethics Statement

The current study was performed by approval of the Ethics Committee of Qazvin Medical University with approval number IR.QUMS.REC.1400.439.

## Peer Review

The peer review history for this article is available at https://www.webofscience.com/api/gateway/wos/peer‐review/10.1002/vms3.70510.

## Data Availability

The data that support the findings of this study are available from the corresponding author upon reasonable request.
